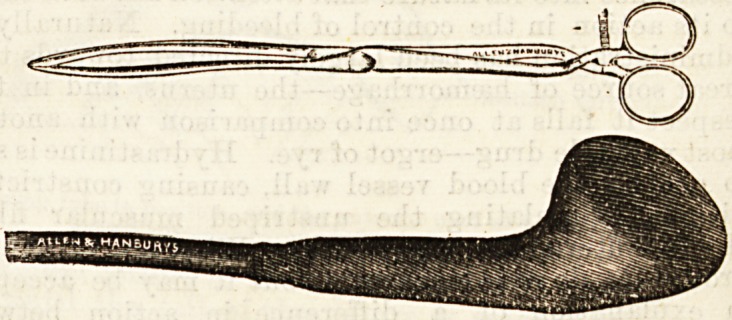# New Appliances and Things Medical

**Published:** 1894-02-03

**Authors:** 


### NEW APPLIANCES AND THINGS MEDICAL.

### PATENT "OZONISED" DIGESTIVE TEA.

(THE UNIVERSAL DIGESTIVE TEA CO., 100, MARKET STREET, MANCHESTER.)

The injurious astringent effects of tannin are well-known, and all our readers will be aware of the fact that it is owing to the unfortunate presence of large quantities of this substance in ordinary infusions of tea that so many persons of weak digestion are obliged to deny themselves the luxury of consuming the otherwise charming and harmless product of the tea-pot. There are two general principles upon which processes for the removal of tannin from tea are based. The first depends upon the fact that tannin forms an insoluble compound with gelatine; and it has more than once been proposed to add gelatine solutions to the tea-pot at the time of making the infusion in order to precipitate the astringent tannates. We do not think, however, that such a plan would commend itself to the average housewife, and moreover we have not found it efficient in practice. The second principle upon which the removal of tannin may be undertaken involves the fact that tannin is very susceptible to processes of oxidation, and is thereby rendered innocuous. In the case of the samples before us it is this latter fact that has been taken advantage of, and the process applied has had a very large measure of success; all that we have examined contain very much less tannin than do ordinary teas of the same quality. The plan adopted, moreover, does not necessitate the addition of chemicals, solid or liquid, to the tea, so that consumers need have no fear of other deleterious materials taking the place of the tannin wh ch has been removed. The following is our analysis of a sample of the *Patent "Ozonised" Digestive Tea*, which is specially recommended by the proprietors for the use of hospitals and institutions. Analysis: Undried sample—total ash, 5·35 per cent.; insoluble mineral matters, 1·60 per cent.; soluble salts, 3·75 per cent.; alkalinity of the ash (expressed as KHO), 2·24 per cent.; total extractives, 31·25 per cent.; tannin (determined by Lœwenthal's method), 6·6 per cent. The tannin in such a tea as this if untreated would have been at least 15 to 20 per cent., and possibly more. The high proportion of soluble salts shows at once that the tea has suffered no treatment which involves previous extraction, and therefore has in no sense lost in strength. But it must be remembered that those who have allowed their palates to become vitiated by getting accustomed to astringent decoctions, may at first miss the very ingredient which it is the object of the proprietors of the "ozonised" teas to remove. That tannin is deleterious is quite certain, and that it is enormously reduced in quantity and so rendered practically harmless in these "ozonised teas" is equally sure.

### FLORADOR FOOD.

(THE FLORADOR FOOD CO., 90, WASHINGTON STREET, GLASGOW.)

This is an excellent preparation of granulated wheaten flour. It yields on analysis: Nitrogen, 2·10 per cent.; mineral matter, 0·246 per cent.; water, 11·920 per cent. The above is a high percentage of nitrogen for a wheat-food so prepared that the whole is in a highly digestible condition, and indicates a high nutritional value. Practical use of the food convinces us of its digestive qualities and general excellence.

### "CHAMPETIER DE RIBES" BAGS.

From Messrs. Allen and Hanbury's we have received a specimen of "Champetier de Ribes" bags. This consists of an inelastic bag covered with rubber, and having a stop-cock tube attached. It is so shaped that when placed *in situ* with the proper forceps (see illustration) and afterwards filled with fluid it fills the lower segment of the uterus, and acts as an artificial "bag of water" in dilating that organ. It has recently been highly spoken of by Dr. Herbert Spencer and others. The specimen forwarded to us, we need hardly state is thoroughly well made and finished.

## Figures and Tables

**Figure f1:**